# Mu desynchronization during observation and execution of facial expressions in 30-month-old children

**DOI:** 10.1016/j.dcn.2016.05.003

**Published:** 2016-05-24

**Authors:** Holly Rayson, James John Bonaiuto, Pier Francesco Ferrari, Lynne Murray

**Affiliations:** a School of Psychology and Clinical Language Sciences, University of Reading, Reading, United Kingdom; b Sobell Department for Motor Neuroscience and Movement Disorders, University College London, United Kingdom; c Dipartimento di Neuroscienze, Università di Parma, Parma, Italy; d Department of Psychology, Stellenbosch University, South Africa

**Keywords:** Mirror neuron system, Facial expression, Mu rhythm, Development, Emotion, Electroencephalography

## Abstract

Simulation theories propose that observing another’s facial expression activates sensorimotor representations involved in the execution of that expression, facilitating recognition processes. The mirror neuron system (MNS) is a potential mechanism underlying simulation of facial expressions, with like neural processes activated both during observation and performance. Research with monkeys and adult humans supports this proposal, but so far there have been no investigations of facial MNS activity early in human development. The current study used electroencephalography (EEG) to explore mu rhythm desynchronization, an index of MNS activity, in 30-month-old children as they observed videos of dynamic emotional and non-emotional facial expressions, as well as scrambled versions of the same videos. We found significant mu desynchronization in central regions during observation and execution of both emotional and non-emotional facial expressions, which was right-lateralized for emotional and bilateral for non-emotional expressions during observation. These findings support previous research suggesting movement simulation during observation of facial expressions, and are the first to provide evidence for sensorimotor activation during observation of facial expressions, consistent with a functioning facial MNS at an early stage of human development.

## 1 Introduction

Facial expressions form an essential component of social interaction, providing us with a base from which we can understand other people’s feelings, or infer their motivations and intentions. As such, accurate recognition and analysis of facial expressions is important for the facilitation of appropriate behaviour within an interaction, and contributes significantly to the success of a social exchange. Emotional facial expression processing is especially important during early development as young children acquire social and communicative skills. Before mastering language, infants understand others’ emotions predominantly via the ‘reading’ of faces (Leppänen and Nelson, 2009), which continues to play a crucial role during social interactions throughout childhood and beyond. The facial expressions of caregivers convey a wealth of information to their offspring during face-to-face exchanges, for example fear or smiling in order to signal the danger or lack thereof posed by a particular object or situation (Klinnert, 1984, Sorce et al., 1985). Furthermore, difficulty recognizing and understanding facial expressions has been associated with a range of adverse child outcomes, including impaired social functioning and behavioural problems (Izard et al., 2001, Leppänen and Hietanen, 2001, Trentacosta and Fine, 2010).

Simulation theories propose that observation of another person performing a facial expression activates the observer’s sensorimotor representations implicated in producing that movement, which aids expression recognition (e.g. Adolphs, 2006). Neurophysiological findings in monkeys have provided the first evidence for such a neural mapping mechanism linking the perception of an action onto its cortical motor representation. These ‘mirror neurons’ were first discovered in the premotor cortex of the macaque monkey (Di Pellegrino et al., 1992), and are a class of neuron that fire both during the execution and observation of a similar action. Consequently, mirror neurons are widely thought to implement a mapping from an observed action to the observer’s motor representation used to perform the same action (Rizzolatti and Craighero, 2004). Evidence from research using a variety of techniques (fMRI, TMS, EEG, depth-electrode recordings) now supports the existence of a homologous mirror neuron system (MNS) in human adults (Iacoboni and Dapretto, 2006, Molenberghs et al., 2012), including the inferior and superior parietal lobules, ventral premotor cortex, and inferior frontal gyrus (IFG), with the superior temporal sulcus (STS) providing the primary visual input. Accordingly, the concept of a human MNS has been suggested as a prospective biological mechanism underlying the perception of facial expressions as proposed by simulation theories, with the observation of another’s action activating like neural processes in the observer as in the performer (Gallese and Sinigaglia, 2011).

Though much mirror neuron research has focused on the study of hand actions, a number of studies have also explored putative MNS involvement in the processing of facial expressions. Indeed, single cell recordings in the ventral premotor cortex of adult macaque monkeys have demonstrated the existence of mirror neurons for facial movements (Ferrari et al., 2003), and a number of fMRI studies with human adults have found common activation of brain areas associated with the MNS during observation, execution, and imitation of facial expressions (Carr et al., 2003, Dapretto et al., 2006, Engell and Haxby, 2007, Hennenlotter et al., 2005, Kircher et al., 2013, Lee et al., 2006, Likowski et al., 2012, Pohl et al., 2013, Van der Gaag et al., 2007). These human studies have demonstrated overlapping activation in response to both static and dynamic facial stimuli (e.g. Carr et al., 2003, Leslie et al., 2004).

Many facial expressions involve both motor and emotional components, and therefore it has been suggested that these aspects are processed by separate, but linked, mirror systems which work together to contribute to facial expression recognition (Van der Gaag et al., 2007). Observation, imitation, and execution of emotional and non-emotional facial expressions result in overlapping patterns of neural activation, with emotional facial expressions eliciting more activation in regions such as the amygdala, insula, and IFG (Carr et al., 2003, Kircher et al., 2013, Van der Gaag et al., 2007, Wicker et al., 2003). It has been proposed that the insula links the frontal component of the MNS with the limbic system, providing a mapping from an observed expression onto internal emotional representations (Dapretto et al., 2006, Rizzolatti et al., 2014).

Despite the work on the MNS providing important information concerning a common neural substrate for emotion observation and execution, its focus exclusively on adult participants leaves open the question of whether such a mechanism is functional from a much earlier age, which would support the hypothesized presence of a simulative process for emotion understanding in the developing brain (Decety and Meyer, 2008). Non-invasive techniques such as EEG are required for studying MNS activity in more challenging populations such as young children and infants. The mu rhythm (8–13 Hz in adults) recorded over the central electrodes, has been identified as an index of MNS activity (Muthukumaraswamy et al., 2004, Pineda, 2008) because it is thought to be generated in the sensorimotor cortex, is modulated during both action execution and observation, and its activity co-varies with BOLD activity in MNS regions during simultaneous EEG and fMRI acquisition (Arnstein et al., 2011). In infancy and early childhood, the mu frequency range is lower than in adults, gradually increasing over time (Marshall et al., 2002). The 6–9 Hz range has been identified as functionally analogous to the adult 8–13 Hz band in early development (Stroganova et al., 1999, Stroganova and Orekhova, 2007), and is considered appropriate for use with children up to 4 years of age (Marshall et al., 2002). As recommended by Cuevas et al. (2014), from here on we refer to mu ‘desynchronization’ where power is significantly decreased from a baseline period, and ‘suppression’ where mu power is significantly different between conditions or regions but not necessarily lower than baseline.

In human adults, the mu rhythm is sensitive to observation and mental imagery of orofacial movements (Muthukumaraswamy et al., 2006, Pfurtscheller et al., 2006, Spiegler et al., 2004), and the few studies that have investigated adult mu activity during observation of emotional facial expressions suggest MNS simulation of facial movements (Cooper et al., 2013, Moore et al., 2012). Interestingly, hemispheric differences in mu activity have been found in during observation of positive and negative facial expressions (Moore et al., 2012), which is in keeping with other research demonstrating the dominance of the right hemisphere for face and emotion processing (Adolphs, 2002, Borod et al., 1998, Killgore and Yurgelun-Todd, 2007 Borod et al., 1998; Killgore and Yurgelun-Todd, 2007).

Around 8–14 months of age, human infants already demonstrate changes in mu rhythm power during observation of hand actions (Marshall and Meltzoff, 2014, Marshall et al., 2011, Nyström et al., 2011, Southgate et al., 2010), but despite the importance of face-face interactions during early childhood (Trevarthen and Aitken, 2001), mu responses to facial expressions in very young populations have not yet been explored. Ferrari and colleagues (Ferrari et al., 2012, Vanderwert et al., 2015) found evidence for MNS involvement during observation and execution of facial gestures in newborn macaque monkeys, with desynchronization demonstrated in the 5–6 Hz EEG rhythm during observation of live human facial gesture performance. This suggests that a functioning MNS could also be present soon after birth in humans, and may play a role in facial expression processing from an early stage in development. Therefore conducting similar studies with younger human populations is now critical in order to address this question.

In the present study, mu rhythm desynchronization in 30-month-olds was explored in response to observation of videos in which adults performed both dynamic emotional and non-emotional facial expressions. While the age group included in this study is particularly difficult for EEG research, it is of importance because of the extensive emotional and social developments that occur during this period (Brownell and Kopp, 2007, Denham, 1998). Children of this age become increasingly adept at reading others’ mental states and emotions (Bartsch and Wellman, 1995; Phillips et al., 2002), and, for example, begin to display more empathic behaviour towards parents (Zahn-Waxler, 1992), and sometimes peers (Nichols et al., 2009, Spinrad and Stifter, 2006). Therefore 30 months constistutes an appropriate age to first explore potential involvement of the sensorimotor system during observation of facial expressions at an early stage in human development. Based on previous studies of hand action observation with young populations and adult EEG studies of facial expressions, we expected to see mu desynchronization during both observation and execution of facial expressions. In keeping with best practices suggested for mu rhythm research with young children (Cuevas et al., 2014), we used dynamic stimuli that included a pre-movement static neutral expression, as well as videos of facial expressions in which the face was scrambled. This enabled comparison of mu power changes relative to a baseline period and a control condition, which allowed us to determine whether any observed effects were simply due to observation of a (the static baseline) face or a face-like stimulus performing meaningless movements (the scrambled condition). Trials in which participants spontaneously produced facial expressions were coded offline and excluded from the main observation analysis. These trials were then analysed separately in lieu of an execution condition.

## 2 Methods

### 2.1 Participants

28 healthy children (15 male, 13 females) aged approximately 30 months took part in this study, which was approved by the University of Reading Research Ethics Committee (21.05.13). Participants were recruited from the Child Development database based in the School of Psychology and Clinical Language Sciences at the University of Reading. Mothers gave written, informed consent before participation. Eleven participants were excluded before analysis due to excessive fussiness/movement during net placement or throughout the experiment (N = 10), and technical difficulties (N = 1), leaving a sample of 17 (10 male, 7 female; age: M = 937.765 days, SD = 44.938). This loss of data is comparable with other EEG studies that have investigated the mu rhythm in young populations (Cannon et al., 2016, Marshall et al., 2013, Southgate et al., 2010).

### 2.2 Stimuli

**Fig. 1:**
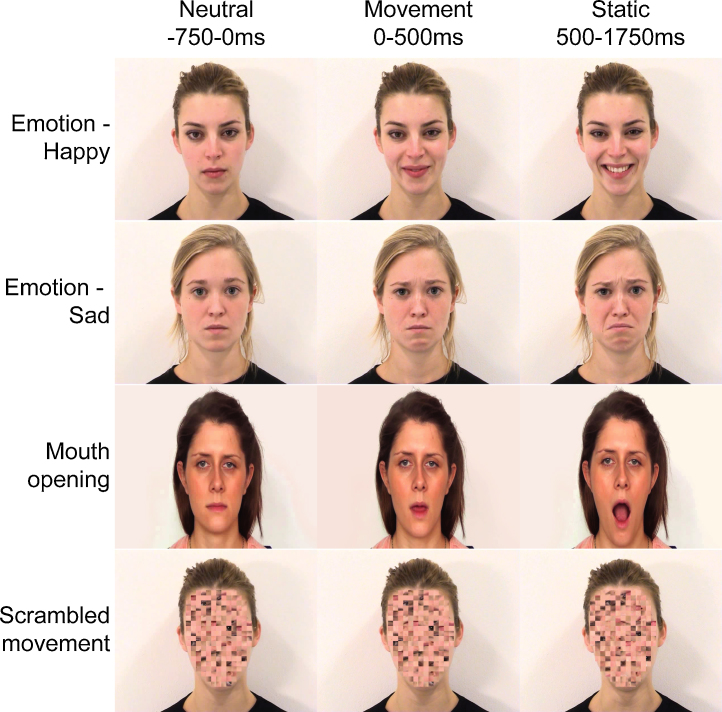
Time-course of stimuli in the four experimental conditions. Each condition included an initial, static neutral expression, followed by a facial movement which lasted approximately 500 ms. After the movement peak, the expression was held for 1250 ms.

Stimuli consisted of short videos (2.5 s) of female actors executing a number of facial expressions. There were four different conditions included in the experiment: a positive condition, ‘happy’; a negative condition, ‘sad’; a non-emotional condition, ‘mouth opening’; and a control condition consisting of scrambled versions of the other videos (i.e. a scrambled version of each happy, sad and mouth opening video). Previous studies have utilized static or non-biological moving stimuli in control conditions (Ferrari et al., 2012); however, we chose to use the scrambled stimuli in order to control for low-level visual features and overall motion across all experimental conditions. The scrambled versions of each video were produced by dividing the face region into square blocks (18 × 18 pixels), randomly shuffling these blocks in the first frame of the video, and then applying the same transformation to each subsequent frame. This resulted in a video with comparable low-level visual and motion features as the original, but with an incoherent movement (see Fig. 1). The videos featuring positive and negative facial expressions were taken from the Amsterdam Dynamic Facial Expression Set (ADFES), which has been well validated in previous research (Van der Schalk et al., 2011). Ratings of the mouth-opening videos on a scale of −2 (negative) to +2 (positive) by a panel of 20 adults confirmed that they represented non-emotional facial expressions (M = −0.10, SD = 0.07). These videos were made comparable with the ADFES stimuli in terms of onset, duration of movement, size, brightness, contrast, and spatial frequency. All videos started with 750 ms of a static/neutral facial expression, followed by 500 ms of movement, and 1250 ms held at the movement peak (Fig. 1).

### 2.3 Procedure

Children were seated on mothers’ laps approximately 65 cm from a computer monitor. Stimuli were presented on the monitor using PsychoPy v1.80.04 (Peirce, 2008) in blocks of 6 video clips of the same facial expression (happy, sad, mouth opening or scrambled; 2 actors per block, 3 videos each). These clips were randomized within blocks, and blocks themselves were pseudo-randomized so that the same condition could not be presented more than twice in succession. The inter-stimulus interval was randomized between 800 and 1200 ms. The experiment was terminated if the child became too inattentive, distressed, moved excessively, or once they had viewed 6 blocks of each condition.

### 2.4 Data acquisition

EEG was recorded using a 128-channel Hydrocel Geodesic Sensor Net (EGI, Corp., Eugene, OR). Data were sampled at 250 Hz with an analogue band-pass filter of 0.1–100 Hz, and were recorded with the vertex as a common reference. Impedances were kept below 50 kΩ. An experimental block began when triggered manually by an experimenter who was watching the participant on a screen from another section of the room. Trial blocks were triggered as soon as the child was attentive to the monitor. Synchronous video recordings of the experiment were also examined offline to allow exclusion of trials in which the child was inattentive, and to enable execution of facial expressions to be coded.

### 2.5 Behavioural coding

In order to identify trials in which participants executed the facial expressions presented during experimental blocks, expressions (happy, sad and mouth opening) were coded offline from the video recordings. All videos were coded by a research assistant blind to the experimental condition being presented. Videos were viewed in real-time and frame-by-frame to accurately identify onsets and offsets of movements. A second independent researcher coded a random 20% of the videos to establish inter-rater reliability, with good reliability obtained (time-unit *ĸ* = 0.86–0.88, event *ĸ* = 0.83).

### 2.6 EEG pre-processing and analysis

After viewing the video recordings and marking periods of inattention using EGI software (NetStation v4.3.1; Electrical Geodesics, Inc., Eugene, OR), EEG data were exported and analysed using the EEGLAB v13.3.2. toolbox (Delorme and Makeig, 2004). Data were bandpass filtered at 2–35 Hz. Epochs ranging from 750 ms before stimulus movement onset to 1750 ms after movement onset from each trial were extracted. Epochs that contained previously marked periods of inattention and epochs in which more than 15% of channels exceeded +/− 250 μV were excluded. A natural-gradient logistic infomax independent component analysis (ICA) was performed on the data (the runica algorithm; Delorme and Makeig, 2004) to decompose the EEG mixed signals into their underlying neural and artefactual components (such as eye and muscle movements). Artefact components were identified and removed using the ADJUST algorithm (v1.1; Mognon et al., 2011). Finally, data were re-referenced to the average of all electrodes.

To compare power relative to baseline in the mu band, we computed event related spectrums (ERSs) for each condition using built-in EEGLAB procedures. Time-frequency decompositions were computed with a fast Fourier transform using a 1-s Hanning window with 50% overlap in 1 Hz bins from 2 to 30 Hz. To make our results comparable with those of other studies, we converted log spectral power to absolute power, and averaged across the 6–9 Hz bins (corresponding to the mu range typically used in sensorimotor system research with young participants: e.g. Cannon et al., 2016, Marshall et al., 2013, Saby et al., 2012). We then computed event-related desynchronization (ERD) as the percentage change of the average absolute power over a 0–750 ms time window (from the onset of facial movement in experimental stimuli until 250 ms after the peak of the full expression) from the condition-specific baseline averaged over −650 ms to −50 ms (prior to the onset of the observed facial movement; Pfurtscheller and Aranibar, 1979). To confirm the suitability of the 6–9 Hz band for use in this study, we calculated ERD during execution trials in the 6–9 Hz and 10–13 Hz (which covers the corresponding adult range) bands. There was indeed greater mu ERD in the 6–9 Hz band (see Supplementary Material), with only ERD in this band significantly lower than baseline. We therefore used this frequency range for the rest of the analyses.

**Fig. 2:**
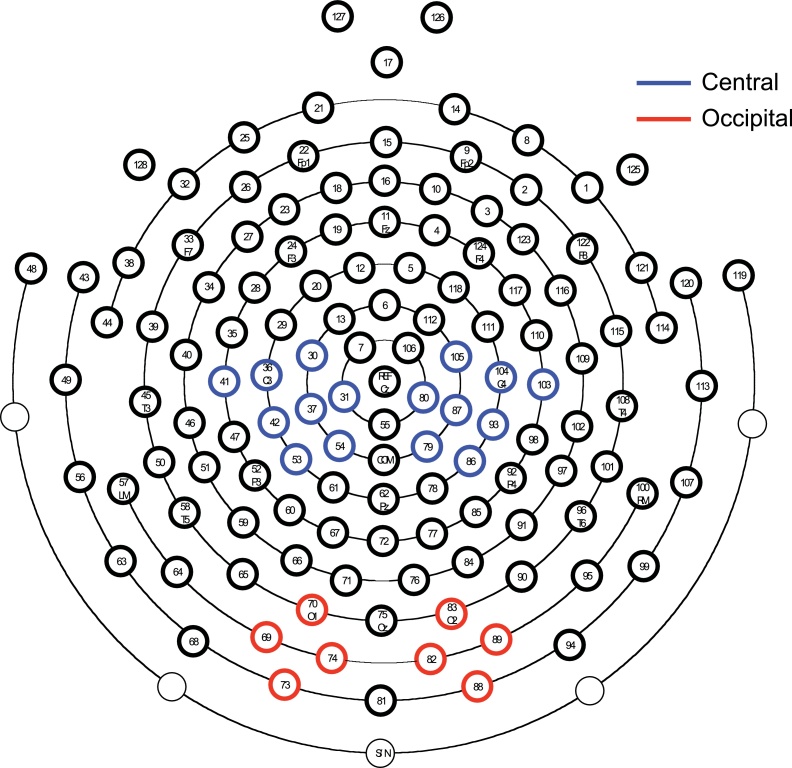
Location of channels included in the central (blue) and occipital (red) clusters (for interpretation of the references to colour in this figure legend, the reader is referred to the web version of this article)..

ERD was calculated for four clusters of electrodes. These were comprised of two central clusters (left and right hemisphere, 8 electrodes each) located around standard C3 and C4 sites for mu rhythm recording, and two occipital clusters (left and right hemisphere, 4 electrodes each) located around standard O1 and O2 sites to control for visual alpha responses (Fig. 2; Umilta’ et al., 2012). For each cluster, in each experimental condition, the ERD values were calculated for each subject.

## 3 Results

In the following analyses, the α-level was set at 0.05 and all post-hoc tests were Bonferroni corrected. The Greenhouse-Geisser correction of degrees of freedom was used when the sphericity assumption was violated (indicated by ε).

### 3.1 Observation trials

To investigate changes in mu power during observation of experimental stimuli, trials marked during behavioural coding as containing execution of happy or sad expressions, or mouth opening movements were excluded. A minimum of 5 trials per condition was required for children to be included in the analysis, which is in keeping with other research (Cannon et al., 2016). This left a total of 15 participants with an average of 56.477 trials (SD = 16.357) overall (happy, M = 13.867, SD = 5.208; sad, M = 14.933, SD = 4.818; mouth opening, M = 13.067, SD = 6.703; scrambled = 14.600, SD = 4.256). Before comparing conditions and clusters to each other, we wished to establish whether desynchronization indeed occurred relative to the baseline period. Significant mu desynchronization was found in the left central cluster for mouth opening [M = −31.043, SD = 17.975; *t*(14) = −6.689, *p* < 0.001], but not for any other condition [all *p* > 0.400]. In the right central cluster, there was significant mu desynchronization for mouth opening [M = −26.203, SD = 17.254; *t*(14) = −5.882, *p* < 0.001], happy [M = −15.164, SD = 14.780; *t*(14) = −3.974, *p* = 0.001], and sad [M = −28.327, SD = 12.393; *t*(14) = −8.852, *p* < 0.001] conditions, with significant mu *synchronization* in the right central cluster for the scrambled condition [M = 2.549, SD = 3.282; *t*(14) = 3.008, *p* = 0.009]. There was no significant mu desynchronization in either occipital cluster relative to baseline [all *p* > 0.200], except for mu desynchronization in O2 for the sad condition [M = −15.421, SD = 20.262; *t*(14) = −2.948, *p* = 0.001].

**Fig. 3:**
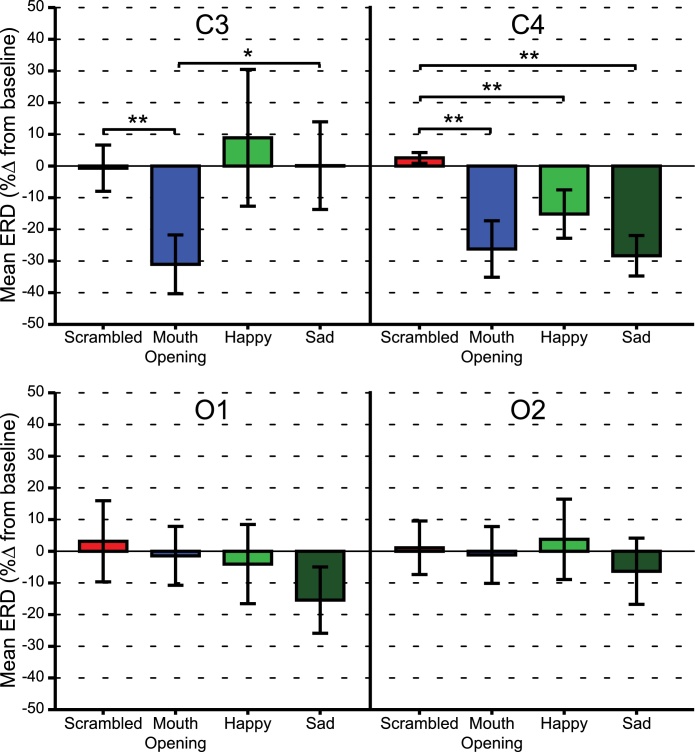
ERD for each condition in central (top) and occipital (bottom) clusters. Error bars represent the mean +/− standard error, *p < 0.05, **p < 0.005. In the left central cluster, ERD in response to mouth opening was significantly greater compared to scrambled and sad conditions, and in the right central cluster, ERD was significantly greater in all conditions compared to scrambled. There was no significant difference across conditions in O1 or O2.

Having established the presence of mu desynchronization, a 2 × 2 × 4 repeated-measures ANOVA was conducted, with region (central/occipital), hemisphere (left/right) and condition (happy/sad/mouth opening/scrambled) as within-subject variables. The ANOVA revealed a significant main effect of region [*F*(1, 14) = 14.223, *p* = 0.002, $\eta_{p}^{2}$ = 0.504] and of condition [*F*(3, 42) = 5.764, *p* = 0.002, $\eta_{p}^{2}$ = 0.292]. These results were qualified by significant region × hemisphere [*F*(1, 14) = 10.301, *p* = 0.006, $\eta_{p}^{2}$ = 0.424] and region × condition [*F*(3, 42) = 6.048, *p* = 0.002, $\eta_{p}^{2}$ = 0.302] interactions. A significant three-way region × hemisphere × condition interaction [*F*(1.813, 25.380) = 6.298, *p* = 0.007, $\eta_{p}^{2}$ = 0.310, ε = 0.604] was also revealed, which was followed up by conducting two separate repeated-measures ANOVAs for each region (central/occipital, Fig. 3).

The analysis of central clusters revealed significant main effects of both hemisphere [*F*(1, 14) = 7.717, *p* = 0.015, $\eta_{p}^{2}$ = 0.355] and condition [*F*(1.617, 22.638) = 10.723, *p* = 0.001, $\eta_{p}^{2}$ = 0.434, ε = 0.539], and a significant hemisphere × condition interaction [*F*(2.091, 29.275) = 6.108, *p* = 0.006, $\eta_{p}^{2}$ = 0.304, ε = 0.697]. Pairwise comparisons demonstrated that mu ERD was not significantly different in the left and right hemisphere for scrambled [*t*(14) = −0.866, *p* = 0.401] and mouth opening conditions [t(14) = −1.346, *p* = 0.200], but was significantly greater in the right hemisphere for happy [t(14) = 2.193, *p* = 0.046] and sad conditions [t(14) = 3.437, *p* = 0.004]. In the left hemisphere, ERD in response to mouth opening was significantly greater compared to scrambled [*t*(14) = 6.011, *p* < 0.001] and sad conditions [*t*(14) = −3.818, *p* = 0.011] (and approached significance for happy [*t*(14) = *p* = 0.057]), and in the right hemisphere, ERD was significantly greater in all conditions compared to scrambled (mouth opening [*t*(14) = 6.778, *p* < 0.001]; happy [*t*(14) = 4.416, *p* = 0.004]; sad [*t*(14) = 9.346, *p* < 0.001]).

The analysis of occipital clusters revealed no significant main effects of hemisphere [*F*(1, 14) = 1.397, *p* = 0.257, $\eta_{p}^{2}$ = 0.091] or condition [*F*(3, 42) = 1.719, *p* = 0.178, $\eta_{p}^{2}$ = 0.109], and there was no significant hemisphere × condition interaction [*F*(3,42) = 0.882, *p* = 0.458, $\eta_{p}^{2}$ = 0.059]. This indicates that mu desynchronization was specific to central clusters and not due to changes in occipital alpha power.

### 3.2 Execution trials

To explore changes in the mu band while executing rather than observing facial expressions, separate analyses were conducted for participants who performed happy, sad or mouth opening expressions during the experiment. There were not enough instances of each expression to analyse separately; therefore we collapsed across expression type. This left 11 participants with a minimum of 5 execution trials each (M = 17.000, SD = 9.945; per participant).

**Fig. 4:**
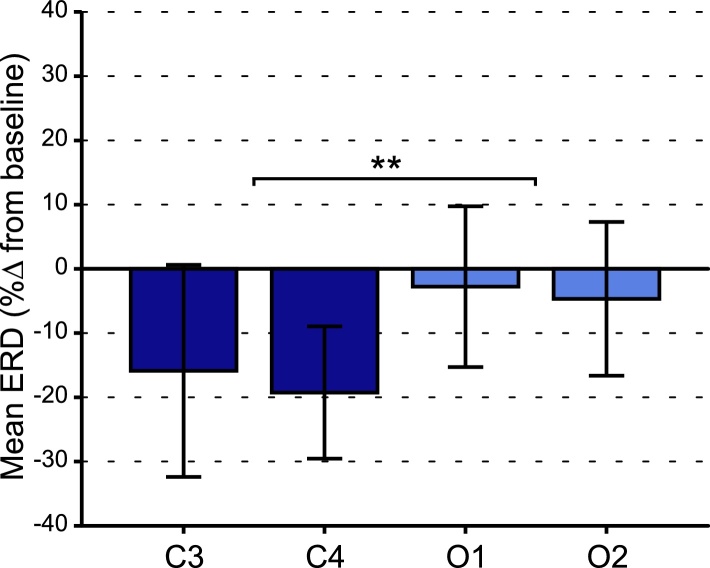
ERD in central and occipital clusters during movement execution. Error bars represent the mean +/− standard error, *p < 0.05, **p < 0.005. There was relatively greater mu suppression in central compared to occipital clusters.

For the coded execution trials (M = 11.647, SD = 10.891; per participant), significant mu desynchronization was found relative to baseline in the right central cluster [M = −19.258, SD = 17.063; *t*(10) = −3.743, *p* = 0.004], but not for any other cluster [all *p* > 0.080]. To explore differences in mu ERD during execution of facial expressions, a 2 × 2 repeated-measures ANOVA was conducted, with region (central/occipital) and hemisphere (left/right) as within-subject variables (Fig. 4). The ANOVA revealed a significant main effect of region [*F*(1, 10) = 6.048, *p* = 0.034, $\eta_{p}^{2}$ = 0.377], with relatively greater mu suppression in central [M = −17.567, SD = 19.624] compared to occipital clusters [M = −3.717, SD = 19.601].

## 4 Discussion

Findings from monkeys and adult humans suggest that the MNS is involved in the processing of facial expressions (Carr et al., 2003, Ferrari et al., 2003, Moore et al., 2012, Van der Gaag et al., 2007), but previous research has not explored whether the human MNS for faces is functional from an early stage in development. The results of the present study suggest activation of the sensorimotor system during both observation and execution of facial expressions in children as young as 30 months of age, which corroborates evidence from adult studies implicating the MNS in the simulation of facial expressions.

Specifically, we used EEG to determine whether mu desynchronization occurs when children observe positive, negative, and neutral dynamic facial expressions. Our main finding was that there was significant mu ERD in central clusters in response to all facial expressions during observation relative to a static neutral face, apart from the scrambled condition. Significant mu suppression (and desynchronization in the right hemisphere) was also demonstrated over central electrodes during execution of emotional and non-emotional expressions. Interestingly, whereas the effect during observation was bilateral in central clusters for mouth opening expressions, significant mu ERD during observation of happy and sad facial expressions was found only in the right hemisphere.

As well as being the first study to show mu desynchronization during observation of facial expressions early in childhood, the present study extends previous EEG studies of the facial MNS by comparing emotional and non-emotional facial expressions. Additionally, most studies of mu rhythm activity use either observation of static stimuli or non-biological movement as control conditions (e.g. Ferrari et al., 2012, Moore et al., 2012), and thus do not address the specificity of the EEG response to biological movements (Cuevas et al., 2014). A recent meta-analysis on the mu rhythm has strongly recommended the use of multiple control conditions in order to assess EEG response specificity for the investigation of the MNS (Fox et al., 2016). Our use of a static neutral face baseline period controlled for observation of a face alone, and as the movement of low-level facial features was still visible in the scrambled condition, this controlled for observation of meaningless biological movement. The lack of significant mu desynchronization in response to the observation of scrambled facial expressions demonstrates that the significant mu ERD seen in the other conditions is not simply due to observation of a moving face-like stimulus or other attentional factors. Additionally, the lack of mu ERD in occipital regions during facial expression observation demonstrates that the effect seen in central clusters is not a result of alpha desynchronization in visual cortex, but is specific to somatomotor cortical regions.

Our finding that mu desynchronization was right lateralized during observation of emotional expressions is in line with many studies showing right hemisphere dominance for emotional facial processing (Adolphs et al., 1996, Calvo and Beltrán, 2014, De Haan and Nelson, 1998, Moreno et al., 1990). Bilateral activation of human MNS areas during action-observation has often been reported (for a review see Rizzolatti et al., 2014), however most MNS studies have investigated observation of hand actions, and therefore may not be directly comparable with our study. In fact, other EEG studies of the facial MNS have demonstrated differential mu responses to emotional facial expressions (Moore et al., 2012), and to faces associated with reward performing happy expressions (Gros et al., 2015) in the right hemisphere. Right lateralized ERPs have also been found during emotional facial expression discrimination in the somatosensory cortex, which is where the alpha mu rhythm is thought to be generated (Sel et al., 2014). In infants, EEG studies have shown the right hemisphere to be more sensitive to early emotional experience with caregivers (Bowers and Heilman, 1984, De Haan et al., 2004), including exposure to maternal depression (Dawson et al., 1992, Jones et al., 2009), and consistent with our results, right lateralized ERPs have been found in children during observation of static facial expressions (Batty and Taylor, 2006, De Haan et al., 2004, Field et al., 1998). Our results suggest that right lateralized sensorimotor activity during observation of emotional faces is in place by 30 months of age. It could be that an MNS for facial expressions is active in even younger children and infants, and it would interesting to investigate whether a lateralized response to emotional faces develops over time as infants form and strengthen associations between motor and emotional representations.

Changes in mu rhythm activity during observation of facial expressions might also, at least in part, be explained by covert imitation. In adults, the observation of facial expressions leads to subtle, measurable effects at the muscle level, similar to covert facial responses (i.e. facial mimicry; Dimberg et al., 2002, Dimberg, 1982, Lundqvist and Dimberg, 1995). It is possible that in our study children displayed such responses, but they were not detectable at the behavioural level. In other words, although our fine-grained behavioural analysis allowed us to remove any trials containing overt movements, the EEG responses described during observation trials may still partly reflect the synergy between observing and imitating facial expressions. Results from a very recent electromyography (EMG) study (Geangu et al., 2016) do suggest that the primary muscle involved in smiling (the zygomaticus major) is activated during observation of happy faces in three-year-old children. The authors interpret this as evidence for a perception-action matching mechanism facilitated by an MNS for facial expressions. However, one MEG study has shown that mu rhythm modulation can occur without significant facial EMG activity, and therefore decreases in mu power may not necessarily reflect covert imitation (Nishitani and Hari, 2002). Further research is clearly required to explore any relationship between mu rhythm responses in children and imitative covert responses.

Although our results imply sensorimotor system involvement in facial expression processing, they do not give any indication of whether children explicitly recognized the expressions they observed. Explicit and implicit recognition of facial expressions are thought to be distinct processes (Mathersul et al., 2009), involving separate but overlapping networks of brain regions (Adolphs, 2002, Habel et al., 2007). Explicit recognition is the volitional mapping of an observed facial expression onto a discrete category with an associated label, such as ‘happiness’ or ‘sadness’. On the other hand, implicit recognition involves the automatic activation of representations associated with a facial expression, including emotional and motor components (e.g. the ‘feeling’ of happiness and the motor commands used to smile). Investigating the explicit recognition of facial expressions in young populations with limited verbal capacities is very difficult, however, it has been shown that by three years of age, children do begin to accurately name expressions (Pons et al., 2004). This implies that children start to explicitly recognize certain facial expressions around this age, but measures such as naming may rely on additional abilities that are still developing. By 30 months of age, and indeed much earlier (Farroni et al., 2007), children are capable of producing and show implicit recognition of all basic facial expressions, including those used in this study (Leppänen and Nelson, 2009). There are many event-related EEG studies that support implicit recognition of various facial expressions in infancy, which includes differentiation between emotional and neutral expressions (Leppänen et al., 2007, Taylor-Colls and Pasco Fearon, 2015, De Haan et al., 2004), as well as observational research showing that young children modulate their behaviour in response to the emotional versus neutral expressions of others (Nichols et al., 2010). Therefore, although we did not test explicit recognition in this study, children of this age do appear to implicitly recognize a number of facial expressions, and the differential mu desynchronization we found in response to emotional and non-emotional facial expressions suggests a role for the sensorimotor system in this process.

One limitation of the present study is the lack of an explicit execution or imitation condition as it is difficult to instruct young children to perform such a task. Nevertheless, there were enough spontaneous instances of infant happy, sad, and mouth opening expression production to combine them into an execution condition, with mu suppression demonstrated in central compared to occipital clusters.

To summarise, we found that in 30 month old children, significant mu rhythm desynchronization occurred during observation and execution of emotional and non-emotional facial expressions compared to static neutral faces, but not during observation of meaningless biological movement of a face-like stimulus. There was significant mu desynchronization in the left and right hemispheres during observation of non-emotional expressions, but desynchronization was right lateralized for emotional expressions, consistent with the concept of right hemisphere dominance in emotional face processing. These findings suggest activation of the sensorimotor system during observation and execution of facial expressions from an early stage in human development, which is consistent with simulation theories of facial expression processing involving a MNS.

## Funding

This work was supported by a Medical Research Council UK doctoral studentship (MR/J003980/1) awarded to Holly Rayson.

## References

[bib0005] Adolphs R., Damasio H., Tranel D., Damasio A.R. (1996). Cortical systems for the recognition of emotion in facial expressions. J. Neurosci..

[bib0010] Adolphs R. (2002). Recognizing emotion from facial expressions: psychological and neurological mechanisms. Behav. Cogn. Neurosci. Rev..

[bib0015] Adolphs R. (2006). How do we know the minds of others? Domain-specificity, simulation, and enactive social cognition. Brain Res..

[bib0020] Arnstein D., Cui F., Keysers C., Maurits N.M., Gazzola V. (2011). μ-suppression during action observation and execution correlates with BOLD in dorsal premotor, inferior parietal, and SI cortices. J. Neurosci..

[bib0025] Bartsch K., Wellman H. (1995). Children Talk About the Mind.

[bib0030] Batty M., Taylor M.J. (2006). The development of emotional face processing during childhood. Dev. Sci..

[bib0035] Borod J.C., Cicero B.A., Obler L.K., Welkowitz J., Erhan H.M., Santschi C., Whalen J.R. (1998). Right hemisphere emotional perception: evidence across multiple channels. Neuropsychology.

[bib0040] Bowers D., Heilman K.M. (1984). Dissociation between the processing of affective and nonaffective faces: a case study. J. Clin. Neuropsychol..

[bib0045] Brownell C., Kopp C. (2007). Socioemotional Development in the Toddler Years: Transitions and Transformations.

[bib0050] Calvo M.G., Beltrán D. (2014). Brain lateralization of holistic versus analytic processing of emotional facial expressions. Neuroimage.

[bib0055] Cannon E.N., Simpson E.a., Fox N.a., Vanderwert R.E., Woodward A.L., Ferrari P.F. (2016). Relations between infants’ emerging reach-grasp competence and event-related desynchronization in EEG. Dev. Rev..

[bib0060] Carr L., Iacoboni M., Dubeau M.-C., Mazziotta J.C., Lenzi G.L. (2003). Neural mechanisms of empathy in humans: a relay from neural systems for imitation to limbic areas. Proc. Natl. Acad. Sci. U.S.A..

[bib0065] Cooper N.R., Simpson A., Till A., Simmons K., Puzzo I. (2013). Beta event-related desynchronization as an index of individual differences in processing human facial expression: further investigations of autistic traits in typically developing adults. Front. Hum. Neurosci..

[bib0070] Cuevas K., Cannon E.N., Yoo K., Fox N. (2014). The infant EEG mu rhythm: methodological considerations and best practices. Dev. Rev..

[bib0075] Dapretto M., Davies M.S., Pfeifer J.H., Scott A.a., Sigman M., Bookheimer S.Y., Iacoboni M. (2006). Understanding emotions in others: mirror neuron dysfunction in children with autism spectrum disorders. Nat. Neurosci..

[bib0080] Dawson G., Klinger L.G., Panagiotides H., Hill D., Spieker S. (1992). Frontal lobe activity and affective behavior of infants of mothers with depressive symptoms. Child Dev..

[bib0085] De Haan M., Nelson C., Slater A. (1998). Discrimination and categorisation of facial expressions of emotion during infancy. Perceptual Development: Visual, Auditory and Speech Perception in Infancy.

[bib0090] De Haan M., Belsky J., Reid V., Volein A., Johnson M.H. (2004). Maternal personality and infants’ neural and visual responsivity to facial expressions of emotion. J. Child Psychol. Psychiatry Allied Discip..

[bib0095] Decety J., Meyer M. (2008). From emotion resonance to empathic understanding: a social developmental neuroscience account. Dev. Psychopathol..

[bib0100] Delorme A., Makeig S. (2004). EEGLAB: an open source toolbox for analysis of single-trial EEG dynamics including independent component analysis. J. Neurosci. Methods.

[bib0105] Denham S. (1998). Emotional Development in Young Children.

[bib0110] Di Pellegrino G., Fadiga L., Fogassi L., Gallese V., Rizzolatti G. (1992). Understanding motor events: a neurophysiological study. Exp. Brain Res..

[bib0115] Dimberg U., Thunberg M., Grunedal S. (2002). Facial reactions to emotional stimuli: automatically controlled emotional responses. Cogn. Emotion.

[bib0120] Dimberg U. (1982). Facial reactions to facial expressions. Psychophysiology.

[bib0125] Engell A.D., Haxby J.V. (2007). Facial expression and gaze-direction in human superior temporal sulcus. Neuropsychologia.

[bib0130] Farroni T., Menon E., Rigato S., Johnson M.H. (2007). The perception of facial expressions in newborns. Eur. J. Dev. Psychol..

[bib0135] Ferrari P.F., Gallese V., Rizzolatti G., Fogassi L. (2003). Mirror neurons responding to the observation of ingestive and communicative mouth actions in the monkey ventral premotor cortex. Eur. J. Neurosci..

[bib0140] Ferrari P.F., Vanderwert R.E., Paukner A., Bower S., Suomi S.J., Fox N.A. (2012). Distinct EEG amplitude suppression to facial gestures as evidence for a mirror mechanism in newborn monkeys. J. Cogn. Neurosci..

[bib0145] Field T., Pickens J., Fox N.A., Gonzalez J., Nawrocki T. (1998). Facial expression and EEG responses to happy and sad faces/voices by 3-month-old infants of depressed mothers. Br. J Dev. Psychol..

[bib0150] Fox N., Bakermans-Kranenburg M., Koo K., Kiernan L., Cannon E., Vanderwert R., van IJzendoorn M. (2016). Evaluation of the EEG mu-rhythm as an index of human mirror neuron activity: a meta-analysis. Psychol. Bull..

[bib0155] Gallese V., Sinigaglia C. (2011). What is so special about embodied simulation?. Trends Cogn. Sci..

[bib0160] Geangu E., Quadrelli E., Conte S., Croci E., Turati C. (2016). Three-year-olds’ rapid facial electromyographic responses to emotional facial expressions and body postures. J. Exp. Child Psychol..

[bib0165] Gros I.T., Panasiti M.S., Chakrabarti B. (2015). The plasticity of the mirror system: how reward learning modulates cortical motor simulation of others. Neuropsychologia.

[bib0170] Habel U., Windischberger C., Derntl B., Robinson S., Kryspin-Exner I., Gur R.C., Moser E. (2007). Amygdala activation and facial expressions: explicit emotion discrimination versus implicit emotion processing. Neuropsychologia.

[bib0175] Hennenlotter A., Schroeder U., Erhard P., Castrop F., Haslinger B., Stoecker D., Ceballos-Baumann A.O. (2005). A common neural basis for receptive and expressive communication of pleasant facial affect. Neuroimage.

[bib0180] Iacoboni M., Dapretto M. (2006). The mirror neuron system and the consequences of its dysfunction. Nat. Rev. Neurosci..

[bib0185] Izard C., Fine S., Schultz D., Mostow A., Ackerman B., Youngstrom E. (2001). Emotion knowledge as a predictor of social behavior and academic competence in children at risk. Psychol. Sci..

[bib0190] Jones N.A., Field T., Almeida A. (2009). Right frontal EEG asymmetry and behavioral inhibition in infants of depressed mothers. Infant Behav. Dev..

[bib0195] Killgore W.D.S., Yurgelun-Todd D.A. (2007). The right-hemisphere and valence hypotheses: could they both be right (and sometimes left)?. Soc. Cogn. Affect. Neurosci..

[bib0200] Kircher T., Pohl A., Krach S., Thimm M., Schulte-Rüther M., Anders S., Mathiak K. (2013). Affect-specific activation of shared networks for perception and execution of facial expressions. Soc. Cogn. Affect. Neurosci..

[bib0205] Klinnert M.D. (1984). The regulation of infant behavior by maternal facial expression. Infant Behav. Dev..

[bib0210] Lee T.-W., Josephs O., Dolan R.J., Critchley H.D. (2006). Imitating expressions: emotion-specific neural substrates in facial mimicry. Soc. Cogn. Affect. Neurosci..

[bib0215] Leppänen J., Hietanen J. (2001). Emotion recognition and social adjustment in school-aged girls and boys. Scand. J. Psychol..

[bib0220] Leppänen J., Nelson C. (2009). Tuning the developing brain to social signals of emotions. Nat. Rev. Neurosci..

[bib0225] Leppänen J.M., Moulson M.C., Vogel-Farley V.K., Nelson C.A. (2007). An ERP study of emotional face processing in the adult and infant brain. Child Dev..

[bib0230] Leslie K.R., Johnson-Frey S.H., Grafton S.T. (2004). Functional imaging of face and hand imitation: towards a motor theory of empathy. Neuroimage.

[bib0235] Likowski K.U., Mühlberger A., Gerdes A.B.M., Wieser M.J., Pauli P., Weyers P. (2012). Facial mimicry and the mirror neuron system: simultaneous acquisition of facial electromyography and functional magnetic resonance imaging. Front. Hum. Neurosci..

[bib0240] Lundqvist L.-O., Dimberg U. (1995). Facial expressions are contagious. J. Psychophysiol..

[bib0245] Marshall P.J., Meltzoff A.N. (2014). Neural mirroring mechanisms and imitation in human infants. Philos. Trans R. Soc. Lond. B Biol. Sci..

[bib0250] Marshall P.J., Bar-Haim Y., Fox N.A. (2002). Development of the EEG from 5 months to 4 years of age. Clin. Neurophysiol..

[bib0255] Marshall P.J., Young T., Meltzoff A.N. (2011). Neural correlates of action observation and execution in 14-month-old infants: an event-related EEG desynchronization study. Dev. Sci..

[bib0260] Marshall P.J., Saby J.N., Meltzoff A.N. (2013). Imitation and the developing social brain: infants’ somatotopic EEG patterns for acts of self and other. Int. J. Psychol. Res..

[bib0265] Mathersul D., Palmer D.M., Gur R.C., Gur R.E., Cooper N., Gordon E., Williams L.M. (2009). Explicit identification and implicit recognition of facial emotions: II. Core domains and relationships with general cognition. J. Clin. Exp. Neuropsychol..

[bib0270] Mognon A., Jovicich J., Bruzzone L., Buiatti M. (2011). ADJUST: an automatic EEG artifact detector based on the joint use of spatial and temporal features. Psychophysiology.

[bib0275] Molenberghs P., Cunnington R., Mattingley J.B. (2012). Brain regions with mirror properties: a meta-analysis of 125 human fMRI studies. Neurosci. Biobehav. Rev..

[bib0280] Moore A., Gorodnitsky I., Pineda J. (2012). EEG mu component responses to viewing emotional faces. Behav. Brain Res..

[bib0285] Moreno C.R., Borod J.C., Welkowitz J., Alpert M. (1990). Lateralization for the expression and perception of facial emotion as a function of age. Neuropsychologia.

[bib0290] Muthukumaraswamy S.D., Johnson B.W., McNair N.A. (2004). Mu rhythm modulation during observation of an object-directed grasp. Brain Res. Cogn. Brain Res..

[bib0295] Muthukumaraswamy S.D., Johnson B.W., Gaetz W.C., Cheyne D.O. (2006). Neural processing of observed oro-facial movements reflects multiple action encoding strategies in the human brain. Brain Res..

[bib0300] Nichols S.R., Svetlova M., Brownell C.A. (2009). The role of social understanding and empathic disposition in young children’s responsiveness to distress in parents and peers. Cogn. Brain Behav. Interdiscip. J..

[bib0305] Nichols S.R., Svetlova M., Brownell C.A. (2010). Toddlers’ understanding of peers' emotions. J. Genet. Psychol..

[bib0310] Nishitani N., Hari R. (2002). Viewing lip forms: cortical dynamics. Neuron.

[bib0315] Nyström P., Ljunghammar T., Rosander K., von Hofsten C. (2011). Using mu rhythm desynchronization to measure mirror neuron activity in infants. Dev. Sci..

[bib0320] Peirce J.W. (2008). Generating stimuli for neuroscience using PsychoPy. Front. Neuroinf..

[bib0325] Pfurtscheller G., Aranibar A. (1979). Evaluation of event-related desynchronization (ERD) preceding and following voluntary self-paced movement. Electroencephalogr. Clin. Neurophysiol..

[bib0330] Pfurtscheller G., Brunner C., Schlögl A., Lopes da Silva F.H. (2006). Mu rhythm (de)synchronization and EEG single-trial classification of different motor imagery tasks. Neuroimage.

[bib0335] Phillips A.T., Wellman H.M., Spelke E.S. (2002). Infants’ ability to connect gaze and emotional expression to intentional action. Cognition.

[bib0340] Pineda J. a. (2008). Sensorimotor cortex as a critical component of an extended mirror neuron system: does it solve the development, correspondence, and control problems in mirroring?. Behav. Brain Funct..

[bib0345] Pohl A., Anders S., Schulte-Rüther M., Mathiak K., Kircher T. (2013). Positive facial affect − an fMRI study on the involvement of insula and amygdala. PLoS One.

[bib0350] Pons F., Harris P.L., de Rosnay M. (2004). Emotion comprehension between 3 and 11 years: developmental periods and hierarchical organization. Eur. J. Dev. Psychol..

[bib0355] Rizzolatti G., Craighero L. (2004). The mirror-neuron system. Annu. Rev. Neurosci..

[bib0360] Rizzolatti G., Cattaneo L., Fabbri-Destro M., Rozzi S. (2014). Cortical mechanisms underlying the organization of goal-directed actions and mirror neuron-based action understanding. Physiol. Rev..

[bib0365] Saby J.N., Marshall P.J., Meltzoff A.N. (2012). Neural correlates of being imitated: an EEG study in preverbal infants. Soc. Neurosci..

[bib0370] Sel A., Forster B., Calvo-Merino B. (2014). The emotional homunculus: ERP evidence for independent somatosensory responses during facial emotional processing. J. Neurosci..

[bib0375] Sorce J.F., Emde R.N., Campos J.J., Klinnert M.D. (1985). Maternal emotional signaling: its effect on the visual cliff behavior of 1-year-olds. Dev. Psychol..

[bib0380] Southgate V., Johnson M.H., El Karoui I., Csibra G. (2010). Motor system activation reveals infants’ on-line prediction of others' goals. Psychol. Sci..

[bib0385] Spiegler A., Graimann B., Pfurtscheller G. (2004). Phase coupling between different motor areas during tongue-movement imagery. Neurosci. Lett..

[bib0390] Spinrad T.L., Stifter C.A. (2006). Toddlers’ empathy-related responding to distress: predictions from negative emotionality and maternal behavior in infancy. Infancy.

[bib0395] Stroganova T., Orekhova E., de Haan M. (2007). EEG and infant states. Infant EEG and Event-related Potentials.

[bib0400] Stroganova T., Orekhova E., Posikera I. (1999). EEG alpha rhythm in infants. Clin. Neurophysiol..

[bib0405] Taylor-Colls S., Pasco Fearon R.M. (2015). The effects of parental behavior on infants’ neural processing of emotion expressions. Child Dev..

[bib0410] Trentacosta C.J., Fine S.E. (2010). Emotion knowledge, social competence, and behavior problems in childhood and adolescence: a meta-analytic review (Oxford, England). Soc. Dev..

[bib0415] Trevarthen C., Aitken K.J. (2001). Infant intersubjectivity: research, theory, and clinical applications. J. Child Psychol. Psychiatry Allied Discip..

[bib0420] Umilta’ M.A., Berchio C., Sestito M., Freedberg D., Gallese V. (2012). Abstract art and cortical motor activation: an EEG study. Front. Hum. Neurosci..

[bib0425] Van der Gaag C., Minderaa R.B., Keysers C. (2007). Facial expressions: what the mirror neuron system can and cannot tell us. Soc. Neurosci..

[bib0430] Van der Schalk J., Hawk S.T., Fischer A.H., Doosje B. (2011). Moving faces, looking places: validation of the Amsterdam dynamic facial expression set (ADFES). Emotion (Washington, D. C.).

[bib0435] Vanderwert R.E., Simpson E.a., Paukner A., Suomi S.J., Fox N. a., Ferrari P.F. (2015). Early social experience affects neural activity to affiliative facial gestures in newborn nonhuman primates. Dev. Neurosci..

[bib0440] Wicker B., Keysers C., Plailly J., Royet J., Gallese V., Rizzolatti G., Garnier A.T. (2003). Both of us disgusted in my insula: the common neural basis of seeing and feeling disgust. Neuron.

[bib0445] Zahn-Waxler C. (1992). Development of concern for others. Dev. Psychol..

